# Supplementary material for: Exploring the Psychological Impacts of COVID-19 Social Restrictions on International University Students: A Qualitative Study
Source: Int J Environ Res Public Health. 2022 Jun 22;19(13):7631. doi: 10.3390/ijerph19137631 (PMC9266157; doi:10.3390/ijerph19137631)
Supplement: Supplementary file 1 [file ijerph-19-07631-s001.zip › Supplementary file S1.pdf]

**Supplementary file S1.** Consolidated criteria for reporting qualitative studies (COREQ): 32-item checklist

| Topic and Item No. | Guide Questions/Description | Response |
|---|---|---|
| **Domain 1: Research team and reflexivity** | | |
| *Personal Characteristics* | | |
| 1. Interviewer/facilitator | Which author/s conducted the interview or focus group? | Amani Al-Oraibi, Lauren Fothergill, Mehmet Yildirim |
| 2. Credentials | What were the researcher's credentials? E.g. PhD, MD | PhD researchers |
| 3. Occupation | What was their occupation at the time of the study? | Research Associates (Health Sciences) |
| 4. Gender | Was the researcher male or female? | Female (AA, LF), Male (MY) |
| 5. Experience and training | What experience or training did the researcher have? | Mixed methods researchers, experienced interviewers and focus group moderators, GCP trained |
| *Relationship with participants* | | |
| 6. Relationship established | Was a relationship established prior to study commencement? | Researchers met the participants during recruitment |
| 7. Participant knowledge of the interviewer | What did the participants know about the researcher? e.g., personal goals, reasons for doing the research | Participants knew that the interviewers were university researchers. They knew that the interviewers were not involved in the delivery of the asymptomatic testing service or RB-TPP scheme. |
| 8. Interviewer characteristics | What characteristics were reported about the interviewer/facilitator? e.g., Bias, assumptions, reasons, and interests in the research topic | Participants knew that AA, LF and MY and LF were interested in the experiences of students during the COVID-19 pandemic. |
| **Domain 2: Study design** | | |
| *Theoretical framework* | | |
| 9. Methodological orientation and theory | What methodological orientation was stated to underpin the study? e.g. grounded theory, discourse analysis, ethnography, phenomenology, content analysis | Thematic analysis |
| Participant selection | | |
| 10. Sampling | How were participants selected? e.g. purposive, convenience, consecutive, snowball | Purposive sampling |
| 11. Method of approach | How were participants approached? e.g. face-to-face, telephone, mail, email | Participants were approached and recruited by email |
| 12. Sample size | How many participants were in the study? | Students: 29 across 8 focus groups.<br>Staff: 17 interviewed. |
| 13. Non-participation | How many people refused to participate or dropped out? Reasons? | No participants actively withdrew. |
| *Setting* | | |
| 14. Setting of data collection | Where was the data collected? e.g., home, clinic, workplace | Data were collected online (video-conferencing platform) |
| 15. Presence of non-participants | Was anyone else present besides the participants and researchers? | No |

| | | |
|---|---|---|
| 16. Description of sample | What are the important characteristics of the sample? e.g., demographic data, date | Students: Nationalities, living circumstances (on/off campus), and experiences of COVID-19 (i.e., those who had recently tested positive for COVID-19, or never tested positive), and specifically targeted students who had experienced at least one period of self-isolation.<br><br>Staff: to represented diversity of occupational role and nature of student contact. Specifically, staff were invited to participate if they held a role providing direct support for international students, provided accommodation support, mental health support or other stu-dent-facing services, or worked in another role with a responsibility for pastoral care (e.g., academic tutor or supervisor). |
| *Data collection* | | |
| 17. Interview guide | Were questions, prompts, guides provided by the authors? Was it pilot tested? | Yes. The questioning guide was pilot tested with students/staff who were not participants in this study. |
| 18. Repeat interviews | Were repeat interviews carried out? If yes, how many? | No repeat interviews. |
| 19. Audio/visual recording | Did the research use audio or visual recording to collect the data? | Interviews and focus groups were audio-recorded using a video-conferencing platform. Interviewers kept cameras on to assist with establishing rapport. |
| 20. Field notes | Were field notes made during and/or after the interview or focus group? | Yes. |
| 21. Duration | What was the duration of the interviews or focus group? | Focus groups ranged from 58 to 90 minutes (mean: 68.8 minutes). Staff interviews were 29 to 69 minutes (mean: 44.5 minutes). |
| 22. Data saturation | Was data saturation discussed? | Yes. |
| 23. Transcripts returned | Were transcripts returned to participants for comment and/or correction? | No – due to time constraints (rapid project to inform service provision during the pandemic). |
| **Domain 3: analysis and findings** | | |
| *Data analysis* | | |
| 24. Number of data coders | How many data coders coded the data? | Two. |
| 25. Description of the coding tree | Did authors provide a description of the coding tree? | No, however initial coding was informed by the interview guide, and coding was continuously refined. |
| 26. Derivation of themes | Were themes identified in advance or derived from the data? | These were derived from the data. |
| 27. Software | What software, if applicable, was used to manage the data? | N'Vivo |

| | | |
|---|---|---|
| 28. Participant checking | Did participants provide feedback on the findings? | No – due to time constraints (rapid project to inform service provision during the pandemic). |
| *Reporting* | | |
| 29. Quotations presented | Were participant quotations presented to illustrate the themes / findings? Was each quotation identified? e.g., participant number | Yes. |
| 30. Data and findings consistent | Was there consistency between the data presented and the findings? | Yes. |
| 31. Clarity of major themes | Were major themes clearly presented in the findings? | Yes. |
| 32. Clarity of minor themes | Is there a description of diverse cases or discussion of minor themes? | Yes. |
