# Supplementary material for: Exploring the Psychological Impacts of COVID-19 Social Restrictions on International University Students: A Qualitative Study
Source: Int J Environ Res Public Health. 2022 Jun 22;19(13):7631. doi: 10.3390/ijerph19137631 (PMC9266157; doi:10.3390/ijerph19137631)
Supplement: Supplementary file 1 [file ijerph-19-07631-s001.zip › Supplementary file S2_topic guides.pdf]

Student Interview / Focus Group Guide
Your views on COVID-19 and student wellbeing

<u>About You (Interview Sample Characteristics)</u>

1. Briefly introduce yourself.

- [age, undergraduate or postgraduate, year of study, what you study, international or home student, living at home, halls, private accommodation (on or off campus), institution]
- **Have you taken part in a university COVID-19 testing programme or had a COVID-19 test elsewhere?**
- **Are you currently self-isolating? If so, why?**
- **Have you isolated more than once?** Find out timeframe during semester for isolation – of particular, note self-isolation during second surge Oct onwards.
- **Were you told to self-isolate after receiving a university asymptomatic test?**
- **Did you go home over the winter break or were you staying in university accommodation during this time?**

<u>Impact of self-isolation</u>

- **What are your views on self-isolation? Is it important, or not?**
- **How did you feel when you learned you needed to isolate? How do you think other students' experiences of self-isolating have been?**
- **How did being in self-isolation impact you? Did your thoughts, feelings, or mood change whilst you were isolating? Or afterwards?** E.g. Do you have any examples that you feel comfortable sharing? Did you have any particular concerns or worries?

<u>Barriers and enablers to self-isolation</u>

- **What are some things that made going into self-isolation easier? What helped you most during isolation?** E.g. Can be emotional or practical supports
- **Were there any challenges to going into isolation? Anything that made it difficult during your time in isolation?** E.g. Again, can be emotional or practical. Explore differences in those who tested positive vs. those who had been in contact with someone with Covid.
- **Did you feel you were able to adhere to the isolation?** If no, why not? What would have helped?
- **What would encourage students to self-isolate? What might discourage students from adhering to self-isolation advice?** Have other students generally adhered to self-isolation? Are there any particular groups that would find it harder than others, and why?

<u>Support needs during isolation</u>

- **What were some practical things that you needed or would have liked during isolation? Can you think of other things that would have been helpful?** E.g. peer support, social activities, communication, organisation and services at the university level.
- **Did you use any services or seek out support for your mental wellbeing during self-isolation? What helped? Are there other supports you think would have**

been helpful?** E.g. using wellbeing apps or online tools, calling the counselling centre.

- **What was the support like from your institution during self-isolation? Was there anything the university did that was really helpful or could have done differently?** e.g. check ins from university staff, personal tutors.
- **How can the university best support students during periods of self-isolation? What are the key things that would be helpful for yourself or others?**

<u>Winter Break and January Return</u>

- **How did you feel about going home for the winter break? How did you feel about the process for returning in January?** Did you have any concerns or worries? Did you understand the process for leaving and return campus? Views on coming back and being on campus again, being tested, their wellbeing or mental health, views on self-isolation if they need to.
- **What are your views on students being tested for COVID-19 before leaving campus for the winter break?** If you had a test, what went well? Were there any particular issues or problems?
- **If you had a test, how did it make you feel?** E.g. were you happy to have it and why? Did you feel safer? Did this impact your mental health, anxiety, perceived risk, self-isolation?
- **What do you think might stop students from taking a test? Is there anything the university could do to encourage students to take a test if offered?**

<u>Vaccinations</u>

- **What are you views about the Covid vaccine? What do you think are the positives? Do you think there are any negatives?** e.g. Who does it benefit? And in what way? Students / staff / local communities / our families / the public / healthcare services / other? Do you think it would change how people feel or behave with regards adhering to social distancing / self-isolation?
- **What would help encourage students to get a vaccine if it is offered to them in the future? What factors might discourage students?** Are there any practical things the university could do to encourage vaccine uptake?

Staff Interview / Focus Group Guide
Your views on COVID-19 and student wellbeing

Name of interviewee: Date:

What is your current job role?

In your role, do you have face-to-face contact with students?

Do you have other types of direct student contact? In what capacity?

Are you connected to the university COVID-19 testing services? Asymptomatic testing programme or on-campus health centre testing (Pillar 2)?

Have you had to self-isolate yourself? Do you have any other experience with COVID-19?

Response, adherence, impacts

How do you think students generally respond when they're told they have to self-isolate?

Do they think students are generally being adherent to self-isolation advice or not? (If yes or no, then why)

Does students' response to, adherence to, or the broader impact of self-isolating differ according to the reason students need to self-isolate (triggered by self or others)?

Does students' response to, adherence to, or the broader impact of self-isolating alter students' engagement with asymptomatic (or other) testing?

How do you think students are impacted during periods of self-isolation? (E.g. physically, logistically, socially, environmentally, mentally etc).

Has any of this changed, or might it change, during different periods of pandemic and with different government restrictions or university regulations? (E.g. early on, through different government restrictions, being at university, leaving for winter break and returning in January).

Mental health and wellbeing

Have you noticed any particular mental health impacts in students who have to self-isolate? Through the pandemic more broadly? Has this raised any particular concerns?

Have you noticed any changes to students' mental health during different periods of pandemic? Might you expect changes in the future? (E.g. early on, through government restrictions, being at university, leaving for winter break and returning in Jan)

Barriers and facilitators:

What do you think can be done to make it easier for students to cope during self-isolation (and adhere to it) - both university-level initiatives in a higher education setting, but also more broadly for supporting young people)?

What factors might discourage students from adhering to self-isolation advice? (For university students and more broadly for young people).

Are there any particular groups that might find it harder to adhere and why? (E.g. high-risk groups, those with mental health concerns, minority group members?)

Support needs:

Do you know what is in place to support students who are self-isolating? What do students tend to access and why?

Which support approaches work and which don't? Why?

Are there any barriers for staff in supporting students who are self-isolating? (E.g. workloads, training, worries about getting COVID, overload of issues arising from COVID, etc)

Are there any barriers for staff in supporting students with mental health concerns?

What else could be done?

Vaccines?

Do you know how students feel about the vaccine? Would they have one if offered? Do you think this would make any difference to their behaviour around self-isolation?

Would students receiving a vaccine make a difference to you in your role? (e.g., student-facing staff feeling they need more COVID protection?)
