# Supplementary material for: Exploring the Psychological Impacts of COVID-19 Social Restrictions on International University Students: A Qualitative Study
Source: Int J Environ Res Public Health. 2022 Jun 22;19(13):7631. doi: 10.3390/ijerph19137631 (PMC9266157; doi:10.3390/ijerph19137631)
Supplement: Supplementary file 1 [file ijerph-19-07631-s001.zip › Supplementary file S3.pdf]

Supplementary file S3: Description of each theme and subtheme, including representative quotes

Subtheme	Quote	Type of participant (Student or staff)
Theme 1: Practical, academic, and psychological challenges faced during self-isolation and the COVID-19 pandemic		
Logistics of self-isolation	I think for me, the challenges I faced coming back to the UK and having to self-isolate was, because I actually moved from a different place last year, I had to plan moving all of my stuff. I had to plan to get groceries, deliver my groceries and everything. I had to unpack and then I had to make sure that I had groceries for the next two weeks. It was quite hectic at that point in time, trying to make sure that everything is done and then I can be comfortable for the next two weeks without leaving the apartment. (Focus group 4, student 1)	Student
	I suppose the challenge might be if you really don't have anyone to help you with shopping or, I don't know, if you get ill a bit you need someone to bring you pills from pharmacy, then you might have to go out. (Focus group 6, student 2)	Student
	For me my biggest challenge was the suddenness. I was given no notice for the second isolation and I had a hard time preparing, so I essentially had to go into isolation with the bare minimum. I didn't have food stocks in my fridge, I didn't have things planned out, you know, so just going it unprepared made it really tough for me. (Focus group 2, student 2)	Student
	I think that the whole online lectures is….this a whole new level. you would think that it might, for some students might be a bit easier because you can re watch it, but I think for a lot of student because it's a little bit like talking to someone on the phone …. It's different than speaking to someone face to face, and you know, if a lecture is kind of in a big room, let's say there can be an echo and you might not hear very well and and again it's the same way, when I was talking about the big event, I think you're more scared, perhaps of asking questions. Because it's literally there for everyone to hear and see you on the screen." (Staff member 16)	Staff
Transition to online learning	I've also mentioned that I think being a design student I guess, usually we have like these studio days where we can just come into the studio and do our work and our tutors would give us feedback. But now we have to jump into Teams at certain time slots to get some feedback, so that's kind of not the same and frankly it's not as effective as I would think. (Focus group 6, student 3)	Student

	Just guidance in terms of self-isolation and placement because we're not really sure what to do still if we miss out any major placement, and then obviously that's a cause of stress. (Focus group 3, student 1)<br><br>Even though I understand, because the school say they hold a certain standard, the same standard, but I feel personally, there's still a bit of difference. There's difference definitely between face-to-face and online lessons. I was hoping that there would be a cut because we still have to pay rent and we have to pay our bills also, which is inclusive of everything. That was a bit sad. I'm sure the government won't help us international students (Focus group 4, student 1)	Student<br><br>Student
Social isolation, loneliness and impacted well-being	I live in Hall as well and the rooms are not really good to be stuck there for 24 into 10 hours. It was not a good experience for me and for this, I might consider moving out or having a vacation again outside of the country unless they lifted up again. It was not a good experience for me emotionally but I was well taken care of by the university and halls. (Focus group 5, student 3)<br><br>what I did was because I was at a new country, towards the end I just ended up calling all my friends each day and just talking to them because I felt quite lonely. (Focus group 1, student 2)<br><br>I didn't really have any outlet to destress since I couldn't meet anybody else, so it was just me in a big house all by myself for two weeks. So I think in general I was just quite tired and down during the isolation period. (Focus group 2, student 3)	Student<br><br>Student<br><br>Student
Theme 2: Coping strategies to self-isolation and life during the pandemic		
I think keeping yourself busy and having someone with you. I think mainly what I did was I called most of my friends or family most of the time, just to let me know what they're doing so that I was engaging in their lives. (Focus group 2, student 4)<br><br>So I think other than having devices to keep me entertained, I think connecting with people out there who are also in the same boat as I was definitely helped a lot, in addition to like support from my family members and basically just self-isolating at home, yeah. (Focus group 3, student 3)<br><br>I had the chance to self-isolated with housemates. I was not alone. We went through this the four of us together, so I think that definitely helped. And, yeah, I think just getting busy with uni and calling my family whenever I could and working out in my room just made things a bit easier. (Focus group 8, student 2)		Student<br><br>Student<br><br>Student
Yeah, it didn't really impact me that negatively as well I think because I was home and I also think because I had something to do, I was really busy with work so I didn't really think too much in the time that I had to myself. But I can imagine if I'm alone		Student

	with my thoughts with nothing to do, that would be quite depressing. So yeah, I kept myself really busy which I think helped a lot. (Focus group 1, student 4)	
	I found it really useful to have streaming things such as Netflix, Amazon Prime, because although I had uni work to do, it had got to some points where I just didn’t feel like doing any work, either because I was mentally feeling a bit down or physically feeling like I didn’t want to work. So being able to just put a movie or put a TV show to get my mind out of it was really useful. (Focus group 8, student 5)	Student
	I guess just programmes like meals delivery and then also movie nights, quiz nights, all those online interactive programmes would help to keep everyone not bored I guess and still keep them busy inside. (Focus group 1, student 1)	Student
Theme 3: Views on further support needed for international students		
Individual ‘check-ins’ with students	I'm not sure for the other school, but for the School of [name], they made it a point where all the teachers kept in touch with us. I thought that was really helpful just to keep things in check, especially academically, and also just to make sure that I don't go insane. (Focus group 4, student 2)	Student
	They were really helpful and everyone was very cordial. They kept checking on me over emails, the welfare officers, and over phone calls from the university COVID team. They were really nice. (Focus group 5, student 3)	Student
	At some point I felt really alone because no-one was like listening to me, but then we have personal tutors and we have a lot of people who we can talk to and stuff, like, I think that’s the good part about it, like, that’s helpful, knowing, like, even if they don't reach out to you, like, you can always talk to your personal tutor or things like that and they’ll try to help you, there’s always someone trying to help, so that made me feel a little better, yeah. (Focus group 7, student 1)	Student
Improved academic support and communication	I would very much like more communication from the [name] school, especially with regard to international and EU students. When we stopped going into placement back in March, we weren’t sure what to do because while everyone else has the comfort of the possibility of going back and forth from their home town, I knew that if I go back to my country I might not be able to come back to the UK because, for example, the borders might close. (Focus group 3, student 1)	Student
	I totally agree that there needed to be better, there needs to be still much better communication from the university to support students going through self-isolation or not, just in general better communication, yeah. (Focus group 8, student 1)	Student

	I think one thing that could be provided is maybe more information, especially when it comes to our assessments. One thing that I've noticed is the university seems to keep saying that they are waiting on the government to make the decisions or they're following other university steps before making a decision which I feel maybe they should come and do what they feel is necessary rather than following what people are doing. Yeah, that would make things clearer. (Focus group 1, student 1)	Student
Inclusive mental health support	I also didn't use any services or seek out any support, just because I didn't really feel like I needed to use them, or a few cases I didn't think that they would help so my issues relating to mental wellbeing. (Focus group 8, student 2)<br><br>I haven't used any of the mental wellbeing support services but I do know that the university really tries to make it known to every student that they do have these services because I have received several mails about the information that these services are available. I just felt at that point that I didn't really need these services yet because I didn't know at what point would I tell myself 'yes, I need help from the university' because even when I did feel kind of depressed early, I would just talk to my flatmates and I would feel better already. (Focus group 1, student 2)<br><br>I also didn't seek help to any support from mental wellbeing and just focused on, yeah, keeping family relations and friend relations going. I also don't think – or maybe I didn't look hard enough – but I wasn't exposed to mental wellbeing support that was attractive to me. (Focus group 8, student 5)	Student<br><br>Student<br><br>Student
